# Effect of Experimental Parameters on Alginate/Chitosan Microparticles for BCG Encapsulation

**DOI:** 10.3390/md14050090

**Published:** 2016-05-11

**Authors:** Liliana A. Caetano, António J. Almeida, Lídia M.D. Gonçalves

**Affiliations:** 1 ESTeSL-Lisbon School of Health Technology, Polytechnic Institute of Lisbon, 1990-096 Lisbon, Portugal; lacaetano@ff.ulisboa.pt; 2 Research Institute for Medicines (iMed.ULisboa), Faculty of Pharmacy, University of Lisbon, 1649-003 Lisbon, Portugal; aalmeida@ff.ulisboa.pt

**Keywords:** alginate, chitosan, BCG, microencapsulation, biocompatibility

## Abstract

The aim of the present study was to develop novel *Mycobacterium bovis* bacille Calmette-Guérin (BCG)-loaded polymeric microparticles with optimized particle surface characteristics and biocompatibility, so that whole live attenuated bacteria could be further used for pre-exposure vaccination against *Mycobacterium tuberculosis* by the intranasal route. BCG was encapsulated in chitosan and alginate microparticles through three different polyionic complexation methods by high speed stirring. For comparison purposes, similar formulations were prepared with high shear homogenization and sonication. Additional optimization studies were conducted with polymers of different quality specifications in a wide range of pH values, and with three different cryoprotectors. Particle morphology, size distribution, encapsulation efficiency, surface charge, physicochemical properties and biocompatibility were assessed. Particles exhibited a micrometer size and a spherical morphology. Chitosan addition to BCG shifted the bacilli surface charge from negative zeta potential values to strongly positive ones. Chitosan of low molecular weight produced particle suspensions of lower size distribution and higher stability, allowing efficient BCG encapsulation and biocompatibility. Particle formulation consistency was improved when the availability of functional groups from alginate and chitosan was close to stoichiometric proportion. Thus, the herein described microparticulate system constitutes a promising strategy to deliver BCG vaccine by the intranasal route.

## 1. Introduction

Enhanced immunization strategies must be urgently found for tuberculosis control [1,2]. The current available vaccine used for pre-exposure vaccination against tuberculosis is *Mycobacterium bovis* BCG. As with most vaccines nowadays, BCG is parenterally administrated by subcutaneous route. This implies a relatively high production cost, the need for cold chain, and the need for trained personnel for vaccine administration, while it also leads to lower patient compliance. Regarding the resulting immune response, parenterally delivered vaccines usually produce poor mucosal responses, which is critical to preventing tuberculosis, as *Mycobacterium* *tuberculosis* normally enters the host through mucosal surfaces. The nasal route might therefore be an attractive alternative administration route [3].

Regarding tuberculosis, it is essential for a new vaccine to better target the lungs while improving interaction with antigen presenting cells (APCs) in the lung mucosa, such as alveolar macrophages [4]. It is also well known that the eradication of *Mycobacterium tuberculosis* with pre-exposure vaccination depends on adequate antigen presentation to amplify the elicited immune response, essentially cellular Th1 types [5,6,7,8]. As such, whole live attenuated bacteria act as the ideal antigen producers and vectors, as they are multigenic and normally mimic pathogens and surpass natural barriers.

In recent decades, several studies have elucidated the pros and cons of the nasal route for vaccine administration. It is well known that, for soluble antigens, limited absorption occurs at the nasal mucosa due to physiological barriers (*i.e.*, mucosal epithelium, rapid mucociliary clearance, protease degradation) [9]. Many strategies have been proposed in order to surpass these barriers and to increase the immunogenicity of intranasal delivered antigens, namely, the use of permeation enhancers, mucosal adjuvants and nano- and microparticulate delivery systems [10,11]. Some studies refer to a boost in the immune response due to an adjuvant effect of particulate delivery systems, combined with the use of potent immunopotentiators, either present in the formulation or co-delivered with antigens [12,13,14,15,16,17,18].

Taking into consideration the aforementioned, it has been hypothesized that BCG bacilli modification through encapsulation in polymeric microparticulate delivery systems could be an alternative to the classical BCG vaccine, suitable for mucosal immunization. Thus, the main goal of this work was to encapsulate whole live BCG into polymers with biocompatible and mucoadhesive properties using only mild conditions, so that BCG viability was maintained and the biocompatibility of the developed microparticulate delivery system was assured. Microencapsulation of BCG in chitosan-alginate microparticles will allow the following to take place *in vivo,* in sequence: bacilli desorption from the particle surface; degradation and erosion of the polymer network; release of bacteria. Moreover, with the entrapment of BCG in polymeric microparticles, it is expected to change the BCG recognition pattern by the immune system and to modulate the mechanism of cellular uptake by APCs cells. The selection of the microsize range was related to the intrinsic length of BCG bacilli rod of approximately 2–4 micrometers, whereas the preference for electropositively charged microparticles depends on their ability to better interact with negatively charged mucin [19,20,21].

The use of biodegradable polymeric particles has been proposed as a promising approach to elicit adequate immune responses, while protecting antigens from degradation [18]. The preparation of polymeric particles can be achieved through a wide range of preparation methods, each one yielding particle formation within a determined size range. For instance, nanoprecipitation and supercritical fluid technology usually yield nanoparticles, whereas spray-drying and solvent evaporation may produce nano- or microparticles depending on the experimental conditions [22,23]. It is generally stated that, for nasal delivery of antigens, nanoparticles are more favorable than particles in the microsize range, as nanoparticles are better taken up by the M-cells present in the nasal associated lymphoid tissue (NALT), and better transported through the epithelial cells (by paracellular and transcellular transference), thus, leading to increased local and systemic immune responses [24,25]. Nevertheless, microparticles sized up to 40 micron have also been described as successful in eliciting immune responses through nasal administration [11,26,27,28,29].

The most commonly described biodegradable polymers are poly(d,l-lactide-co-glycolide) (PLGA) and poly(l-lactide) (PLA); however, particle formation with PLGA and PLA occurs only in the presence of organic solvents. This is a major drawback, for several reasons. Not only the use of organic solvents may lead to relevant toxicological effects, it can also prompt antigen denaturation or hamper cellular vaccine viability, while formulation methods usually require multiple steps and are time consuming. In view of the aim of producing a live vaccine, the longer it takes to carry out the formulation steps, the greater the possibility of losing some of the vaccine or of compromising cell viability, thereby reducing the encapsulation efficiency and potency of the vaccine.

In this context, chitosan (a deacetylated form of chitin extracted from crustaceans), and sodium alginate (a natural product extracted from algae belonging to the Phaeophyceae, mainly species of *Laminaria*) were chosen to prepare polymeric microparticles by ionic cross-linking methods as described elsewhere [30,31,32,33,34,35]. Both chitosan and alginate have been extensively studied as biomaterials and pharmaceutical excipients due to their biodegradability and low toxicity, and have been included in the composition of several foods and dietary supplements [36,37]. With ionic gelation methods, particles are formed in a single step by a simple mechanism, usually involving two different polymers and one complexation agent, by adding one polymer solution to the other one with stirring. Most commonly described complexation agents used with chitosan and alginate are calcium chloride and tripolyphosphate (TPP) [35,38].

The widespread use of polyionic complexation methods presents many advantages, such as their simplicity, versatility and flexibility, being applicable for virtually all polymers which can be polymerized in the presence of a complexation agent, while being easily adjusted by changing a number of experimental parameters. During optimization studies, the formulation conditions can be changed to obtain desired features, namely, particle size, encapsulation efficiency, surface charge, biocompatibility profile, and production yield. The type of used polymers (*i.e.*, chemical nature, molecular weight, viscosity, purity, pH, and other relevant specifications), the polymer to polymer mass ratio, the type and concentration of complexation agent, the homogenization type (*i.e.*, shear, speed and duration) and the polymer to antigen ratio are some of the variables which significantly influence the particles’ characteristics. Furthermore, the mild preparation conditions of these methods allow the encapsulation of antigens without degradation caused by high temperatures, oxidation or hydrolysis, as with other commonly used techniques.

As previously stated, both chitosan and alginate have been extensively used in the preparation of polymeric nano- and micro-particles for immunization purposes. Chitosan and its derivatives are described to increase the absorption of macromolecules through epithelial membranes, and to increase both antigen residence time and uptake at the mucosal site, due to its intrinsic mucoadhesiveness [11,39,40,41,42,43,44]. Chitosan has been used to prepare nano- and microparticles intended for nasal and oral delivery of vaccines with great results, as chitosan particles were able to elicit strong systemic and local immune responses to different antigens [16,24,25,34,41,45,46,47,48,49,50,51,52,53].

Alginates are block copolymers polysaccharides, composed of long homopolymeric regions of mannuronate (M) and guluronate (G), as the result of the conversion of mannuronic and guluronic acid through neutralization during extraction from its natural source. The proportion, distribution and length of these blocks determine the chemical and physical properties of the alginate molecules. While G-blocks provide gel-forming capacity, MM and MG units provide flexibility to the uronic acid chains, with flexibility increasing in the order GG < MM < MG.

Alginates constitute a very versatile material, having numerous pharmaceutical applications due to their gelling, film-forming, thickening and stabilizing properties. It is said that the improved stability of chitosan formulations can be assessed by developing chitosan blends with another polymer, namely sodium alginate [36]. Two other valuable properties of alginates are that they are water-soluble, allowing gel formation without heating or cooling, and also that the alginate matrix allows the entrapment of molecules by capillary forces, which remain free to migrate by diffusion, depending on the size. These features make alginates attractive gelling biopolymers for cell encapsulation purposes. Gel formation and gel structure are determined by alginate type and calcium salt (Ca^2+^), being influenced by pH value, solubility and temperature. For instance, at lower pH values, alginate gel is shrunk and a reduction of the pore size of alginate matrix can be achieved, especially in the case of low G content alginate. As such, these components and factors must be matched in order to optimise the overall formulation of alginate microparticles by ionotropic gelation.

The formulation studies presented in this work aimed the optimization of the preparation conditions of BCG-loaded polymeric particles taking into consideration the final yield of production, encapsulation efficiency, particle size distribution and surface charge. Therefore, variables such as the type of polymer or of polymer blends, polymers solution pH value, the polymer/polymer and BCG/polymers ratio, as well as the type and time of homogenization procedures and order of polymers and counter ions solutions’ incorporation were studied. The herein described effects of experimental conditions on critical features of microparticle formulations provide a processing window for manipulating and optimizing particles in the microsize range for intended applications.

The expected advantages of the herein described systems for vaccine delivery include the capacity of polymeric microparticulate systems to increase antigen residence time (due to the differentiated release profile in the presence of alginate and chitosan) and to enhance antigen interaction with the cell surfaces. Moreover, due to chitosan’s and alginate’s mucoadhesiveness, microparticles would be able to promote mucopenetration, thus increasing antigen delivery.

## 2. Results and Discussion

### 2.1. Characterization of Polymeric Microparticles

The purpose of this study was to optimize the experimental parameters to prepare BCG-loaded polymeric microparticles intended for intranasal immunization studies, presenting suitable size distribution or surface charge, critical aspects for vaccine delivery. Therefore, the conditions for microparticle preparation were optimized during preliminary formulation studies. Two polymers—chitosan and sodium alginate—with different quality specifications (molecular weight, viscosity, G-content, deacetylation degree, purity) were used to prepare plain polymeric microparticles, followed by BCG microencapsulation. The prepared polymeric microparticles were characterized considering particle size distribution, surface charge, morphology, and the final yield of production. FT-IR studies were conducted in order to assess the interaction between chitosan and alginate ionic groups. Particle size was the leading assessed property during formulation optimization studies, oriented towards obtaining microparticles with a mean diameter of 5–10 µm, with a narrow and reproducible size distribution. Another key aspect regarding the preparation of vaccine-loaded polymeric particles is encapsulation efficiency, which should be as high as possible. Biocompatibility of the prepared polymeric microparticles was determined in a cell viability MTT assay, using a human monocyte cell line (THP-1) differentiated into macrophage-like cells, as a model for antigen presenting cells [54,55].

#### 2.1.1. Size Distribution and Surface Charge

Previous studies showed that particle size distribution of plain polymeric microparticles prepared by ionic gelation was greatly influenced by the polymers’ mass ratio and molecular weight [20,56,57]. Therefore, 14 formulations were initially developed with an alginate to chitosan mass ratio (ALG/CS) ranging from 0.02:1 to 4.23:1 (*w*/*w*), according to described Methods I and II, using different combinations of low viscosity (LV) alginate and low molecular weight (LMW) chitosan, medium viscosity (MV) alginate and medium molecular weight (MMW) chitosan, and high viscosity (HV) alginate and high molecular weight (HMW) chitosan. Microparticles were characterised for size distribution (mean diameter and span) and surface charge (zeta potential).

The effect of polymer molecular weight with increasing alginate to chitosan mass ratio on particle size distribution is presented in Table 1. Regarding the use of low molecular weight chitosan, particles ranging from 18 to 34 µm were obtained with a narrow size distribution (span < 2.5) (Table 1). Using chitosan of medium molecular weight yielded a general increase in particle mean diameter, with formulation F13 (0.8:1 alginate to chitosan mass ratio, *w*/*w*) being the only exception. Using chitosan of high molecular weight led to an intermediate particle mean diameter, except also for F13 (Table 1). Herein presented formulations were obtained with a relatively narrow size distribution (span ≤ 5), except for formulation F11 prepared with low molecular weight chitosan (span = 9.5). The obtained span values suggest that particles are formed with better consistency when the availability of the functional groups is close to stoichiometric proportion.

**Figure 1:**
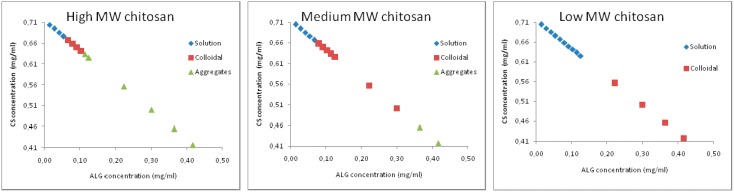
Microparticles domain formation using high, medium and low molecular weight chitosan. The pH of alginate and chitosan solutions was initially set to 4.9 and 4.6, respectively. Three different systems were identified: clear solution (♦), opalescent/colloidal suspension (■), and aggregates (▲).

Broad particle size distributions can be attributed to the presence of larger single particles, which in turn might prompt aggregate formation [58,59]. By visual inspection, we confirmed the presence of aggregates mainly in formulations obtained with polymers of high MW (Figure 1). Best formulations, defined as suitable to yield turbid solution without aggregation, were obtained with chitosan of medium molecular weight when ALG/CS mass ratio ranged from 0.6:1 to 0.12:1, and with chitosan of low molecular weight when ALG/CS mass ratio ranged from 0.4:1 to 1:1.

The obtained results show a greater influence of chitosan molecular weight than alginate to chitosan mass ratio on microparticles size distribution. Overall, the use of chitosan of low molecular weight led to the formation of smaller particles for the majority of ALG/CS mass ratios, resulting in fewer aggregates. This may stem from the ability of chitosan of low molecular weight to diffuse more promptly in the alginate gel matrix to form smaller, more homogeneous particles, whereas, on the contrary, polymers of high molecular weight or viscosity may bind to the surface of such matrices, forming an outer membrane and leading to increment particle size [33,60].

**Figure 2:**
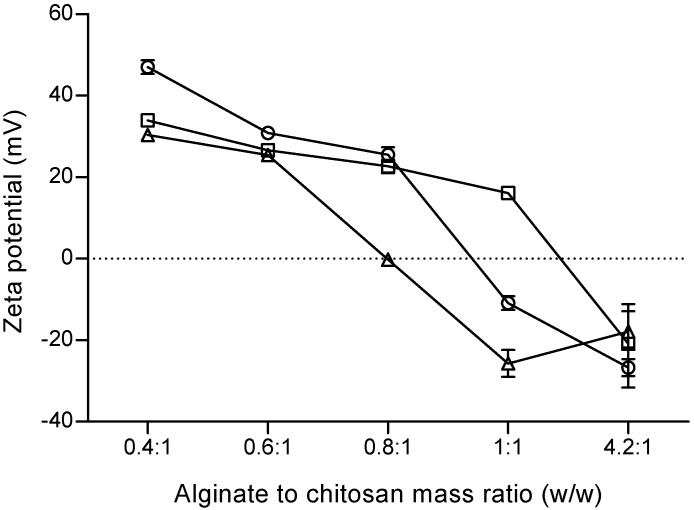
Effect of alginate to chitosan mass ratio on particle surface charge. The pH of alginate and chitosan solutions was initially set to 4.9 and 4.6, respectively. Zeta potential of microparticles prepared with chitosan of low molecular weight (□), medium molecular weight (◌), and high molecular weight (∆). Results are presented as mean ± SD (*n* ≥ 3).

The effect of polymer molecular weight with increasing alginate to chitosan mass ratio on particle surface charge is presented in Figure 2. Alginate to chitosan mass ratios ranging from 0.4:1 to 0.8:1 led to the formation of microparticles with high positive zeta potential values (+22.7 ± 1.6 mV to +47.1 ± 1.7 mV), thus, being positively charged, except for one formulation (F13 prepared with chitosan of high molecular weight) (−0.2 ± 0.8 mV). Higher ALG/CS mass ratios (1:1 and 4.23:1) led to the formation of negatively charged microparticles (−10.9 ± 1.6 mV to −26.7 ± 4.9 mV) with increasing polymer molecular weight. Formulation F14 prepared with chitosan of low molecular weight was the exception (+16.2 ± 0.6 mV).

Zeta potential values provide a quantitative measure of the charge on colloidal particles in liquid suspension. For chitosan-alginate microparticles, surface charge greatly depends on chitosan total protonated amino groups. Zeta potential profiles of ±30 mV are described to prevent aggregation and stabilize particles in suspension [61]. This was also confirmed by visual inspection of the obtained colloidal suspensions, which remained stable without aggregation at room temperature for several days (data not shown).

As for the formulation method, complexation with TPP performed best with 1:1 ALG/CS mass and chitosan of low molecular weight (“F14_Low”), with microparticles presenting a mean diameter of 25.9 ± 0.7 µm, span ≤1.7, and positive surface charge (+22.7 ± 1.6 mV). By using CaCl_2_ as complexation agent, in alternative to TPP, it was possible to improve particle size distribution with 4.23:1 ALG/CS mass ratio and chitosan of low molecular weight (“F0_Low”), with microparticles presenting a reduced mean diameter of 18.5 ± 0.7 µm (span ≤1.4), and negative surface charge (−20.8 ± 7.9 mV).

These results indicate that the molecular weight of the chitosan used to prepare the microparticles had a major impact in particle size distribution, whereas the alginate to chitosan mass ratio had an important role in modulating particle surface charge. It was also possible to identify the conditions which led to a greater heterogeneity in particle formation, evidenced as a broader particle size distribution revealed in increased span values. Overall, it was possible to observe, for microparticles prepared with a given ALG/CS mass ratio, a higher standard deviation of the span when chitosan of medium and high molecular weight were used (with formulation F11 being the exception), thus, indicating that particle size distribution varied considerably and was not completely reproducible. These results were important to put into evidence how to modulate the microparticles size distribution and surface charge profile according to the selected formulation method.

Particle size is determinant in intranasal delivery and mucosal uptake of particles [29], and in the intracellular traffic of the particles [62,63]. Carriers sizing few microns have shown higher potential as intranasal delivery systems of antigens [64,65,66]. As size is increased, which can be partially due to the increase in the sample mass by weight of the microparticles, surface area decreases; this in turn might contribute to a slowdown in the antigen release rate as a depot effect. For the purpose of this study, particle size should be at least 5 µm, in order to enable the entrapment of BCG bacilli, which are short to moderate long rods, 0.3–0.6 × 1–4 µm [67,68]. According to some authors, size must not be greater than 10 µm when phagocytosis is required, with 200 nm to 5 μm being the ideal size [69]. Nevertheless, much larger particles ranging from 1 to 40 μm have been successfully used for intranasal immunization, eliciting good systemic and mucosal responses in mice [9].

Concomitantly with particle size distribution, zeta potential determination allows the estimation of particle suspension stability against subsequent aggregation, as ±30 mV can be an indicator of the particulate systems’ stability [70,71]. Surface charge is a critical parameter that affects the mucoadhesion of chitosan/alginate microparticles to the lung mucosa, which in turn will prolong the residence time of the vaccine at the site of action. The net positive charge indicates the presence of free surface amino groups in F11–F13 in addition to F14 obtained with chitosan of low molecular weight, which will help in initial adhesion to nasal mucosa. Since mucoadhesive properties of chitosan are mainly explained by the electrostatic interaction and by hydrogen bond of amine groups of this cationic polymer with the negatively charged mucin [36], one can expect positively charged particles to be preferable to negatively charged ones.

Taking into consideration the aforementioned results, it was possible to conclude that the association of 4.23:1 ALG/CS mass ratio (formulation F0) and low molecular weight chitosan provided the formulation’s optimal conditions to obtain polymeric microparticles with smaller mean diameter (+18.5 ± 0.7 µm) and narrower particle size distribution (span = 1.4), also with negative surface charge (−20.8 ± 7.9 mV). However, considering that our proposed microparticulate delivery system must be suitable not only to encapsulate whole live BCG bacteria, but also to target the lung mucosa, positively charged particles are expected to be preferable. Therefore, particle size distribution and particle surface charge were considered together, and the 1:1 ALG/CS mass ratio formulation (F14) prepared with chitosan of low molecular weight, with a microparticle size distribution of +25.9 ± 0.7 µm (span = 1.7) and positive surface charge (+16.2 ± 0.6 mV), was chosen to for formulation optimization studies.

##### Effect of Homogenization Method

Preliminary formulation studies showed that particle size distribution of plain polymeric microparticles prepared with the ionic gelation methods was greatly influenced by the type and time of homogenization. Therefore, different homogenization methods were assessed in four different formulations (F0, F12, F13 and F14), in order to obtain microparticles of desired and consistent size distribution. The effect of high-speed homogenization (ultra-turrax, UT) and ultrasonication (US) used for particle preparation with increasing ALG/CS mass ratios of the final formulation is presented in Table 2.

The homogenization by ultrasonication led to the formation of microparticles within a narrower and smaller size range, with mean diameters between 10.8 µm (“F13_Low”) and 14.4 µm (“F14_Low”), and high production yields (>80%) (Table 2). When high-speed homogenization was used, the overall mean diameter of the obtained microparticles greatly increased (Table 2). The use of chitosan of low and high molecular weights resulted in more consistent and reproducible formulation methods, as the size distribution of microparticles with different ALG/CS mass ratios and within the same chitosan molecular weight presented a narrower size distribution, represented by a lower span (Table 2).

Taking into consideration the obtained results, and regarding particle size distribution, method consistency and production yield, we selected formulations “F13_Medium MW chitosan” and “F14_Low MW chitosan” for further optimization studies. In fact, although ultrasonication proved to be effective in the preparation of plain chitosan-alginate microparticles, the ultimate goal was to encapsulate whole live bacilli of BCG. Since both high shear and ultrasonication are said to compromise cell viability, due to the induced cell integrity loss, two alternative homogenization methods were investigated, namely, simple dispersion with a micropipette, or, alternatively, homogenization in an ultrasound water-bath. Increasing homogenization times were evaluated.

Particle mean diameter obtained for both formulations was within the 12.5–21.0 µm range (Table 3). The best results were achieved with simple dispersion (“0 min”) for formulation “F14_Low” (12.5 ± 0.2 µm; −14.9 ± 0.2 mV) and “20 min” in ultrasound water-bath for formulation “F13_Medium” (12.6 ± 0.1 µm; +12.1 ± 0.9 mV). The use of the ultrasound allowed maintaining particle sizes approximated to the desired particle size (10 µm). Particles were overall negatively charged, as five formulations exhibited negative zeta potential values (−49.8 to −14.1 mV), with formulation “F13_Medium” being the one exception.

Particle size increased with increasing homogenization times, as such: from 12.5 to 21.0 µm to “F14_Low” (0 to 20 min); from 16.3 to 19.4 µm to “F13_Medium” (0 to 4 min) (Table 3). Size distribution of microparticles prepared with either 0.8:1 or 1:1 ALG/CS mass ratios, and different chitosan molecular weight, presented with a narrow size distribution (low span) (Table 4), thus indicating a good consistency of the used preparation method. Smaller particle sizes were obtained for F14 formulation at 0 and 4 min, compared to F13, probably due to a more favorable ALG/CS mass ratio, as the stoichiometric proportion of alginate to chitosan of 1:1 might provide a better interaction and nucleation between polymers, leading to smaller sized particles.

##### Effect of Alginate Type and Polymers Addition Order

The results obtained during formulation optimization studies led us towards the rejection of Method (I) and the development of a different preparation method—Method (III). In order to evaluate the effect of the polymers’ specifications on particle size distribution, three different sets of plain microparticles were prepared using three different commercial brands of low viscosity sodium alginate with distinct G-content, namely: low viscosity sodium alginate of high-G content (65%–75%) Protanal™ LF 10/60; ultra-low viscosity sodium alginate of high-G content (63%) Manugel™ LBA; low viscosity sodium alginate of low-G content (40%) Keltone™ LVCR, all approved as pharmaceutical excipient. Chitosan quality specification was kept constant; low molecular weight chitosan with a deacetylation degree of 92% was used. Microparticles were prepared according to two different formulation methods—Methods (II) and (III)—as described (Materials and Methods section) with modifications. The two methods differ in the addition order of the polymers. By changing the polymers’ addition order, it would be possible to modulate the final surface charge of microparticles.

Taking into consideration the aforementioned results obtained for particle size of “F14_Low” prepared by simple dispersion (12.5 ± 0.2 µm), the same homogenization method to prepare these microparticles was used, by simple dispersion with a micropipette for 1 min following additions. In Method (II), chitosan and alginate were allowed to interact prior to TPP addition.

**Figure 3:**
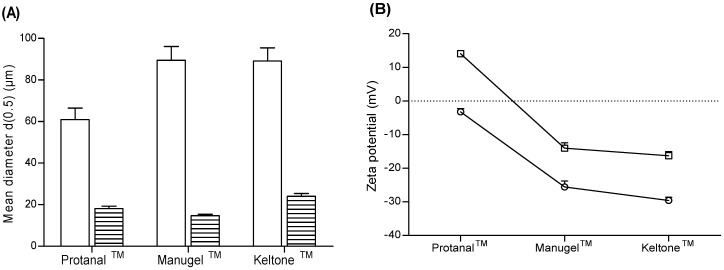
(**A**) Particle size distribution of plain chitosan-alginate microparticles of 1:1 ALG/CS mass ratio, prepared with chitosan of low molecular weight and alginates of decreasing G-content, according to Method (II) (solid) and Method (III) (dashed); (**B**) Zeta potential of plain chitosan-alginate microparticles of 1:1 ALG/CS mass ratio, prepared with chitosan of low molecular weight and alginates of decreasing G-content, according to Method (II) (□) and Method (III) (◌).The pH of alginate, chitosan and TPP solutions was initially set to 6.7, 4.1 and 9.0, respectively. Results are presented as mean ± SD (*n* = 3).

The effect of formulation Methods (II) and (III) with decreasing G-content of the sodium alginate polymers used to prepare microparticles is presented in Figure 3. Regarding particle mean diameter (Figure 3A), microparticles prepared with Method (II) presented a size distribution (d0.5) from 60.9 ± 5.5 µm (Protanal™) to 89.4 ± 6.7 µm (Manugel™). Changing the polymers’ addition order by using Method (III) yielded a particle size distribution with a pronounced decrease in d0.5 values, ranging from 14.7 ± 0.6 µm (Manugel™) to 24.0 ± 1.4 µm (Keltone™). No significant differences were observed between alginates of different G-content within the same formulation method (*p* = 0.4634). As for formulation Methods (II) and (III), it was possible to identify a bimodal particle size distribution depending on the used method. The observed differences were not, however, statistically significant (*p* = 0.1000).

Regarding particle surface charge (Figure 3B), microparticles prepared with Method (II) presented zeta potential values from +14.1 ± 0.6 mV (Protanal™) to −16.3 ± 1.3 mV (Keltone™). Particle surface charge decreased for all formulations when Method (III) was used, reaching negative zeta potential values of −29.6 ± 0.9 mV for Keltone™. Differences among the two evaluated methods were not statistically significant (*p* = 0.4000). Nevertheless, the consistent decrease in zeta potential values suggests that a reorganization of the chitosan-alginate matrix occurred when chitosan was allowed to form a pre-gel with TPP, followed by alginate addition.

These results were important to evidence how the addition order of the polymers plays an important role in the formation of chitosan-alginate microparticles. So far, it seems that ALG/CS mass ratio, homogenization method, and the addition order of the polymers have greater impact on particle size distribution and surface charge than the herein assessed G-content of sodium alginate. Since Method (III) enabled the formation of microparticles with an inferior mean diameter, within a more stable colloidal suspension, it was chosen as the formulation method for the following optimization studies.

##### Effect of pH Value

It is well established that an ionic complex between alginate and chitosan is formed due to interactions between the carboxyl groups of alginate with the amino groups of chitosan [35,56,70,71]. The cationic nature of chitosan (pKa ≈ 6.5) is conveyed by the positively charged –NH_3_^+^ groups, whereas the anionic nature of alginate (pKa ≈ 3.4–3.7) results from the presence of –COO^−^ groups. The cationic nature of chitosan leads to the amino group protonation in acidic to neutral solution, with charge density depending on pH value and chitosan deacetylation degree. These features contribute to the solubility of chitosan in aqueous acidic solutions. Furthermore, it is key for chitosan bioadhesiveness, since chitosan protonated amino groups readily bind to negatively charged surfaces such as mucosal membranes, and for the enhancement of polar drugs transport across epithelial surfaces.

In order to assess the effect of pH value on microparticle formation, several sets of microparticles from formulation “F14_Low” (ALG/CS mass ratio of 1:1, *w*/*w*) were prepared using 1.0 mg/mL solutions of low molecular weight 92% deacetylated chitosan, and low viscosity and high-G content sodium alginate (Protanal™ LF 10/60), with pH value ranging from 3.0 to 7.0. The obtained suspensions were characterized for particle size distribution, surface charge, and yield of production (Table 4).

The use of both alginate and chitosan solutions with a pH value below 5.0 resulted in increased particle size (15 to 44 µm) and particle aggregation (Table 4). Aggregation also occurred when chitosan solution pH was beyond 6.0 (data not shown), due to the loss of chitosan solubility, as chitosan has a pKa value of ≈ 6.5. Considering the desired particle size distribution (*i.e.*, particle mean diameter of approximately 10 µm, and narrow span), the optimal size distributions were obtained when chitosan solution pH value was within 5.0–6.0, and alginate solution pH value within 4.0–6.4, leading to the formation of 10–12 µm sized (d0.5) microparticles. Within this pH range, the carboxyl groups of alginate are ionized, and the amine groups of chitosan are protonated, thus, favouring the optimum interaction for the polyionic complex formation. All these formulations presented a negative particle surface charge (Table 4).

The best system was obtained with formulation final pH value of 5.4, with particle mean diameter of 10.9 ± 0.4 µm, a 2.7 span, and negative surface charge (−18.9 mV) (Table 4). These particles were prepared with chitosan solution at pH = 5.0 and alginate solution pH = 6.4. Although it is well known that the pH-dependent interaction between alginate and chitosan leads to the formation of stronger complexes at a pH value around 4.5–5.0, it is also described that the amine groups of chitosan (pKa ≈ 6.5) have more affinity to alginate mannuronic acid (M) residues (pK_M_ ≈ 3.38) than to guluronic acid (G) residues (pK_G_ ≈ 3.65) [36]. Since a high-G content (≈70%) alginate (Protanal LF™ 10/60) was used, overall alginate pKa was closer to 3.65. This might explain why microparticles of lower mean diameter (10.9 ± 0.4 µm) and narrower size distribution (span = 2.7) were obtained with formulation final pH of 5.4. In fact, at this pH range, the high degree of protonation of chitosan amino groups prompts a significant reaction with alginate carboxyl groups, leading to the formation of stable particles. It would be expected that maximum ionic interaction occurs at a slightly lower pH value for high-M content alginates (such as ≈60% M-content Keltone™ alginate).

The production yield was very low (<17%) for all formulations when determined by gravimetry (Table 4). This is probably related to a low responsiveness of the gravimetric method for the determined mass range, as mass variations occurred within the sub-milligram or micro-range. For that reason, the described method in the Materials and Methods section (Section 3.4.2) based on the quantification of chitosan concentration for the determination of the yield of production of microparticles was selected for further studies. The results obtained were analysed by comparing the different pH conditions.

Regarding zeta potential results, microparticles prepared with the majority of pH combinations were negatively charged (Table 4). This is probably due to the contribution of alginate carboxyl groups to the negative net surface charge, therefore suggesting that the Method (III) provides the arrangement of the polymeric matrix in such way that alginate somehow outers the chitosan particulate core.

It can be observed that particle mean diameter is significantly higher for microparticle suspensions with final pH ≤ 4.3 (Table 4). At this pH value range, alginate approaches its pKa values, and a significant part of alginate starts aggregating and precipitating, which might have contributed to the increased particle mean diameter.

##### Effect of Cryoprotectants Addition

Table 5 summarizes the different batches of plain “F14_Low” microparticles prepared with two concentrations (5% and 10%, *w*/*v*) of three different cryoprotectants. Microparticles were prepared according to Method III with addition of cryoprotectant solution, consisting of sucrose, glucose, or trehalose. Samples were prepared in triplicate. Particle size distribution and zeta potential of samples were assessed for samples without cryoprotectant (batch A) and samples with cryoprotectant (batches B to G), both prior to and after freeze-drying.

The addition of cryoprotectants appears to have contributed to the modification of particle surface charge (Table 5), as microparticles with no cryoprotectant (batch A) presented negative zeta potential values (−19.5 ± 0.7 mV), whereas microparticles prepared with cryoprotectants (batches B, E and F, for 5% sucrose, 10% glucose and 5% trehalose, respectively) presented a positive surface charge, with zeta potential values between +11.6 ± 1.2 mV and +14.9 ± 0.3 mV. This is probably due to the adsorption of the molecules to the surface. It has been described that slightly acidic sucrose and glucose generate a good isotonic medium (in terms of electrostatic stability) for negatively charged particles, but for positively charged particles, as in the case of “F14_Low*”*, these additives reverse zeta potential [72].

Regarding particle mean diameter, it was within micrometer range for all prepared batches (Table 5). With samples analyzed before freeze-drying (on production day), it was possible to observe that there were no significant differences concerning particle size distribution (Table 5). All batches presented similar size distributions, with average d0.5 values of 13.1 ± 0.5 µm, thus, suggesting that addition of cryoprotectants did not influence particle size for batches prepared under thesame conditions.

However, after freeze-drying, particle size distribution profile changed and all batches presented up to 10-fold increased d0.5 values (Table 5), indicating a noteworthy increase of particle mean diameter, probably due to the formation of larger particles or particle aggregates. This could also be seen in the exacerbation of the d0.9 populations for all samples after freeze-drying (data not shown). Nevertheless, the addition of cryoprotectants did prevent some aggregation following freeze-drying, as batch A (particles with no cryoprotectant) presented the highest particle mean diameter, with approximately two-fold higher d0.5 values assigned to batches where cryoprotectant had been added (batches B–G).

As for particle size distribution width, the obtained low span values (2.0 ± 0.5 µm, in average) (Table 5) revealed a high similitude in particle size distribution, thus, suggesting that microparticle preparation was reproducible. Microparticles prepared with 5% and 10% glucose (batches D and E, respectively) performed best, with lower d0.5 values and low span values, thus, corresponding to particles with a smaller, and narrow, particle size distribution.

It can be concluded that microparticle suspensions were affected by the nature and concentration of cryoprotectants, with 10% glucose cryoprotectant (batch E) showing better properties after freeze-drying, with smaller particle size, low span and average zeta potential positive value, compared to microparticles with no cryoprotectant (batch A). Future studies must be conducted with cryoprotectants in order to optimize particle size distribution and surface charge, so that the physicochemical stability of microparticles after freeze-drying can be ensured.

#### 2.1.2. Polymer–Polymer Interaction by FT-IR Analysis

**Figure 4:**
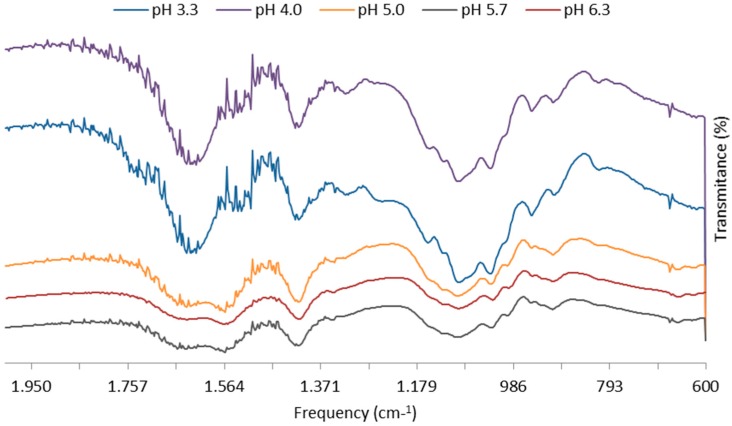
FT-IR spectra of plain chitosan-alginate “F14_Low” microparticles (1:1 ALG/CS mass ratio) with increasing pH of the microparticles suspension. Bands wave numbers (cm^−1^) are as follows: 1641 (amide bond), 1613 (symmetric COO^−^ stretching vibration), 1569 (strong protonated amino peak—from partial N-deacetylation of chitin), and 1415 (asymmetric COO^−^ stretching vibration).

Formation of microparticles of chitosan with alginate is a result of strong interactions by hydrogen bonds between the functional groups of the polymers in which amino and amide groups present in chitosan take part. As a result, there are changes in the FT-IR spectra in the absorption bands of the amino groups, carboxyl groups, and amide bonds [35]. Based on the identification of absorption bands concerned with the vibrations of functional groups present in CS and ALG macromolecules [53], FT-IR analysis was able to illustrate changes in the wave number and absorbance in the region of amino and amide group vibrations with increasing pH of the microparticle suspension (Figure 4).

The FT-IR spectrum of microparticles produced with final pH of 4.0 and 5.7 reveals alginate carboxyl peaks slightly shift from 1613 and 1415 cm^−1^ to 1609 and 1414 cm^−1^, respectively, after complexation with chitosan. Both chitosan peaks were similarly shifted by a few cm^−1^ after complexation with alginate, with the amide peak from 1641 into singlet band at 1609 cm^−1^, and the amino peak from 1559 to 1533 cm^−1^ or 1560 cm^−1^ at pH 4.0 and 5.7, respectively. The observed changes in the absorption bands of the amino groups, carboxyl groups, and amide bonds can be attributed to an ionic interaction between the carbonyl group of alginate and the amino group of chitosan. The peak absorbance of amino groups of chitosan at 1153 cm^−1^ was also present after complexation, thus, suggesting an effective interaction between polymers at pH 4.0 and 5.7.

#### 2.1.3. Surface Morphology

**Figure 5:**
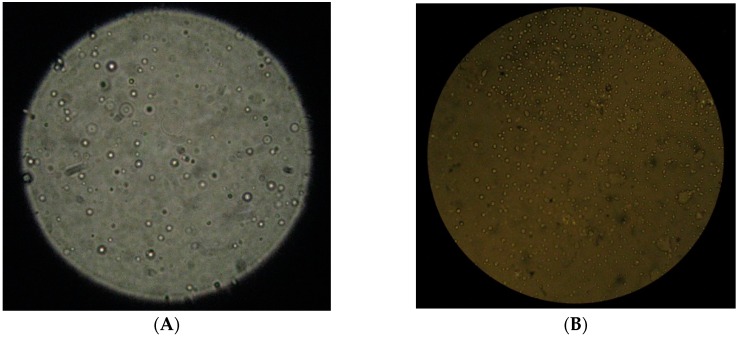
(**A**) Polarized light micrograph (100×) of “F13_Medium” microparticles (0.8:1 ALG/CS) prepared according to Method (III) with chitosan of medium molecular weight; (**B**) Contrast phase micrograph (40×) of “F14_Low*”* microparticles (1:1 ALG/CS) prepared according to Method (II) with chitosan of low molecular weight.

**Figure 6:**
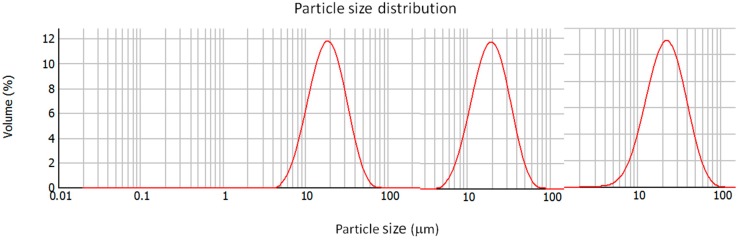
Particle size distribution of microparticles produced with alginate to chitosan ratio of 4.23:1 (F0), 0.8:1 (F13), and 1:1 (F14). F0 microparticles prepared according to Method (I) by alginate ionotropic pre-gelation with CaCl_2_ followed by chitosan coating; F13-F14 microparticles prepared according to Method (III) by chitosan pre-gelation with TPP followed by alginate coating. LMW, low molecular weight chitosan; MMW, medium molecular weight chitosan; HMW, high molecular weight chitosan.

Microparticle morphology was characterized by microscopy. Both F13 and F14 microparticles presented regular and smooth surfaces related to a generic spherical shape (Figure 5). Additionally, particle size distribution observed in microscopic images was consistent with that obtained by laser diffraction, revealing homogeneous populations of narrow particle size distribution (Figure 6).

Considering the results obtained during formulation optimization studies, F14_Low formulation, produced with 1 mg/mL low MW chitosan (pH = 5.0) and 1 mg/mL Protanal™ sodium alginate (pH = 6.4), the ‘simple dispersion’ method was chosen for further BCG encapsulation, so that a suitable formulation of BCG-loaded microparticles can be developed and further assessed in immunization studies. This formulation was chosen because it allowed the formation of microparticles of suitable mean diameter and surface charge without aggregation, under mild conditions and only requiring a few steps, critical for future sterile production during vaccine production.

### 2.2. Encapsulation Efficiency

The ability of chitosan/alginate microparticles to encapsulate *Mycobacterium bovis* BCG depends to a great extent on bacteria surface charge. Therefore, zeta potential of *Mycobacterium bovis* BCG Pasteur and rBCG-GFP strains was measured at low electrolyte concentration. In order to assess the effect of experimental conditions on BCG bacilli surface charge, BCG strains were suspended in different media, whereas BCG previously suspended in 0.9% NaCl was heat killed as it is described in the Materials and Methods section (Table 6).

Both BCG Pasteur and rBCG-GFP bacilli presented predominantly negative zeta potential values (Table 6). Overall, the surface charge of BCG Pasteur appears to be only slightly more electro-negative than rBCG-GFP for all tested conditions. A different macroscopic behavior of cell suspension was also distinguished—BCG Pasteur suspension formed a fluffy surface layer, which led to partial and ephemeral aggregation; this phenomena was not observed for rBCG-GFP strain.

The nature of the adsorbing species on the cell surface of the two strains might explain the obtained variations. The negative surface charge for cells of all *Mycobacterium* BCG species arises from the phosphate groups of phosphodiester linkages between the peptidoglycan and the arabinogalactan of the basic cell wall structure which is common to all species of Mycobacteria [73]. Some hydrophobic interaction involving lipid within the surface may also be involved, since the mycobacterial cell envelope is a lipid-rich, complex structure that surrounds the bacillus and is thought to play a critical role in the pathogenicity of *Mycobacterium tuberculosis*. A large number of mycobacterial lipoproteins have been suggested to be important components for the synthesis of the mycobacterial cell envelope, as well as for sensing processes, protection from stressful factors and host–pathogen interactions [74,75].

Zeta potential profiles showed no major differences between bacilli suspension in either 0.9% NaCl, water, 10 mM PBS, or cell culture medium. However, when BCG bacilli from either strains were suspended in low molecular weight chitosan, an inversion of zeta potential values occurred, in a concentration dependent fashion, suggesting that the mechanism of association of the bacteria to chitosan is, at least partially, mediated by ionic interaction between bacilli and chitosan. Other mechanisms, such as hydrophobic interactions, might also be involved in bacteria microencapsulation.

Taking into consideration the abovementioned, it was hypothesised that the greatest encapsulation/association efficiency for *M. bovis* BCG would be obtained by suspending BCG bacteria in chitosan at a pH below its pKa (e.g., pH = 5), so that the polymer is predominantly positively charged. Additionally, we chose to entrap monodisperse bacteria in chitosan microparticles by means of controlled gelation of chitosan with TPP followed by alginate addition. In this way, a good encapsulation efficiency was sought.

Preliminary formulation studies revealed that particle size distribution and surface charge were influenced by the polymer to polymer mass ratio and the formulation method. Whether BCG microencapsulation would have a great impact on microparticles features was uncertain, thus these parameters were investigated. As such, BCG-loaded “F14_Low” microparticles (1:1 ALG/CS mass ratio) were prepared as described in the Materials and Methods section (Section 3.3.1), by Method (II) or Method (III), with modifications. The encapsulation efficiency was also determined. Table 7 summarizes the different batches of BCG-loaded “F14_Low” microparticles that were prepared and the obtained results.

For microparticles prepared according to Method (II), BCG encapsulation led to decreased particle size in comparison with plain microparticles. In opposition, for microparticles prepared according to Method (III), BCG encapsulation led to increased particle size, referred to as plain microparticles (Table 7). Within BCG-loaded microparticles, particle size increased in a concentration dependent fashion, with increasing BCG loads of 8.3×10^6^, 1.7×10^7^, and 3.3×10^7^ CFU/mL for microparticles prepared by Method (II), and with BCG loads of 8.3×10^6^ and 1.7×10^7^ CFU/mL for microparticles prepared by Method (III) (Table 7).

Regarding particle surface charge, two different patterns were obtained, depending on the formulation method (Table 7). Method (II) produced microparticles (both plain and BCG-loaded) that were electropositively charged, with zeta potential values from +10.5 ± 1.5 mV to +14.1 ± 0.6 mV, whereas Method (III) produced electronegatively charged microparticles (both plain and BCG-loaded), with zeta potential values ranging from −3.2 ± 0.9 mV to −16.4 ± 2.1 mV. In comparison to plain microparticles, BCG-loaded microparticles presented lower zeta potential values regardless of the used formulation method (Table 7). These results indicate that negatively charged BCG bacilli is present, thus, indicating that encapsulation occurred.

The encapsulation of BCG Pasteur into microparticles was efficient (70%–87% E.E.) and occred in a concentration dependent fashion, regardless of the formulation method used (Table 7). The encapsulation mechanism, however, was not determined. Due to the extremely high content of complex lipids present in the BCG cell wall, it is extremely difficult and challenging to achieve efficient, uniform and reproducible microencapsulation experiments. Therefore, we accept that BCG bacilli are sometimes microencapsulated and other times just adsorbed due to partial and irregular adsorption onto the microparticle surface.

### 2.3. BCG Cell Viability

**Figure 7:**
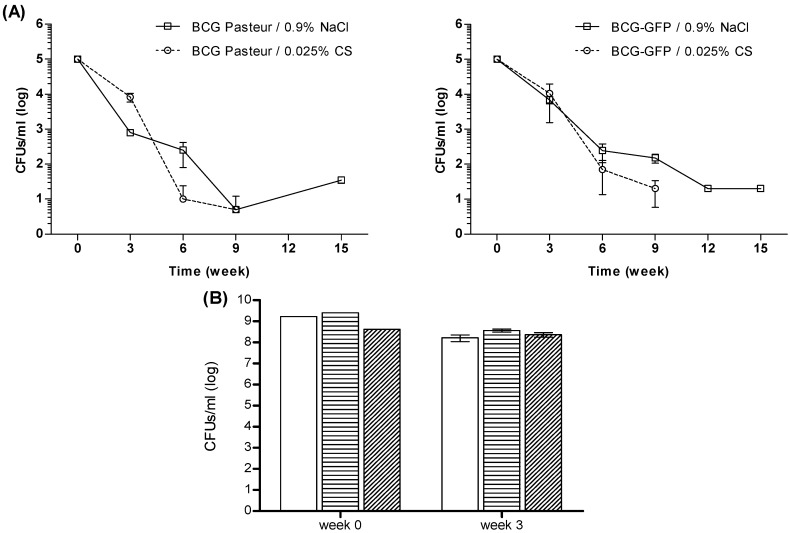
Cell viability of BCG (**A**) After 3 weeks of incubation, agar (Middlebrook 7H10 medium supplemented with OADC) plates inoculated with bacteria of both strains (Pasteur and GFP) that presented a number of colonies of statistical relevance were used to calculate the CFU/mL, by multiplying the colony forming units by the plating factor and the dilution factor. The CFU/mL provides an approximation of the cell density of the original culture. BCG suspension in 0.9% NaCl was used as control; (**B**) BCG Pasteur viability following BCG microencapsulation in “F14_Low” chitosan-alginate microparticles (no fill), BCG suspension in 0.025% low molecular weight chitosan weight (horizontal lines), or BCG suspension in 0.9% NaCl (angled lines). Results are expressed as mean ± S.D.; *n* = 3.

Chitosan is described as having antimicrobial potential [48,49,50]. Whether BCG suspension in chitosan would compromise BCG cell viability was uncertain. Therefore, BCG cell viability following suspension in chitosan was investigated over time by a colony-forming units (CFUs) assay, as described in the Materials and Methods (Section 3.3.1). Both strains BCG Pasteur and rBCG-GFP were assessed in this cell viability study. Results are presented in Figure 7.

Results showed a significant reduction of BCG cell viability for both BCG strains, with small differences (Figure 7A). For rBCG-GFP, viable cell density decreased 1 log on the 3rd week, and approximately 3 log on the 6th week. Regarding BCG Pasteur, cell viability was further reduced, with a viable cell density decrease of 1–2 log on the third week, and approximately 2.5–4 log on the 6th week. These effects were observed for both strains regardless of the suspension media (*p* > 0.05). Overall, although the suspension of BCG in 0.025% chitosan induced a decrease in BCG cell viability, the same effect was observed in the control groups of 0.9% NaCl-suspended BCG. Therefore, chitosan may not be considered cytotoxic at the tested concentration. Figure 7B confirms that viable cell density decreased approximately 1 log after 3 weeks for BCG-loaded chitosan-alginate microparticles and for BCG suspended in 0.025% chitosan. Overall, the microencapsulation procedures preserved BCG integrity and viability, as there were no statistically significant differences (*P* = 0.6314) in cell viability losses between BCG-loaded microparticles and BCG suspended in 0.025% chitosan or in 0.9% NaCl.

### 2.4. In Vitro Cell Viability (MTT Assay)

**Figure 8:**
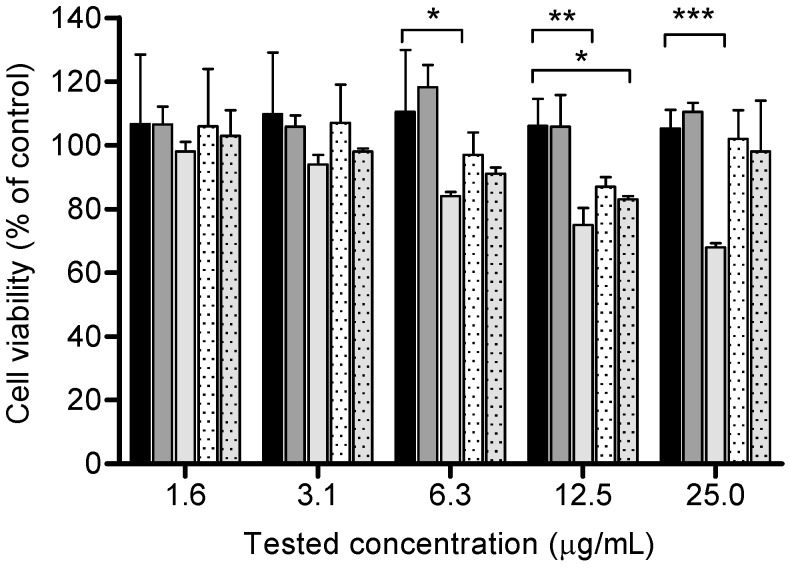
Relative cell viability of THP-1 cell line measured by the MTT reduction. Columns: black—control cells with culture medium; dark grey—BCG-GFP/0.9% NaCl; light grey—BCG-GFP/0.025% LMW chitosan; dotted white—BCG Pasteur/F13_Medium microparticles; dotted grey—BCG Pasteur/F13_High microparticles (1 × 108 CFUs/mL). Results are expressed as mean ± SD (*n* = 3). Statistical differences between the control group and formulations are reported as: *** *p* < 0.001, ** *p* < 0.01, * *p* < 0.05. Cell viability (% of control) = [A] test/[A] control × 100.

The *in vitro* biocompatibility of the microparticles was evaluated with the MTT assay using a PMA-differentiated THP-1 cell line (Figure 8), which is recommended as a model for antigen presenting cells [55].

Results showed no significant reduction of cellular viability after 24 h incubation with chitosan-suspended BCG and BCG-loaded microparticles, except for the highest concentrations of chitosan-suspended BCG, namely, 25 µg/mL (*p* < 0.00001), 12.5 µg/mL (*p* = 0.0077) and 6.3 µg/mL (*p* < 0.05), and a high concentration of BCG-loaded microparticles (12.5 µg/mL). These concentrations exceed concentrations intended for vaccination assays (e.g., 25 µg/mL is about five times higher). Overall, although some formulations induced a slight decrease in cell viability (15%–20%), none of the BCG-loaded microparticles may be considered as cytotoxic since the average values were not significantly different from the control group at tested concentrations. The obtained results are in conformity with other studies where chitosan did not interfere with cell viability [31].

## 3. Materials and Methods

### 3.1. Materials

Sodium alginate polymers of high viscosity (14,000 mPa.s, 20 mg/mL), medium viscosity (3000 mPa.s, 20 mg/mL; M/G ratio of 1.56), and low viscosity (187 mPa.s, 20 mg/mL) (27 mPa.s, 10 mg/mL; M/G ratio of 1.56) were purchased from Sigma-Aldrich (Dorset, UK) (structural viscosity as provided by suppliers). Other tested sodium alginates include: low G-content (40%) Keltone LVCR (218 mPa.s, 20 mg/mL), high G-content (63%) Manugel LBA (773 mPa.s, 100 mg/mL), and high G-content (65%–75%) Protanal LF 10/60 (20–70 mPa.s, 10 mg/mL), which were a gift from FMC BioPolymer A.S. (Sandvika, Norway). Sodium tripolyphosphate (TPP) and calcium chloride (CaCl_2_) were purchased from Sigma-Aldrich (Dorset, UK). Alginate stock solutions were prepared in ultra-purified water.

Most experiments were performed using solutions containing chitosan of low-molecular weight (<150 kDa) and deacetylation degree of 92%; chitosan of medium molecular weight (<450 kDa) and deacetylation degree of 85%; and chitosan of high molecular weight (≈600 kDa) or high structural viscosity (748 mPas, 1% in acetic acid 1%, 20 °C) and undefined deacetylation degree, all purchased from Sigma–Aldrich (Dorset, UK) (specifications as provided by suppliers). The molecular weights were not verified. Chitosan stock solutions were prepared in 1% acetic acid solution in ultra-purified water.

The BCG strains—*M. bovis* BCG Pasteur strain 1173 (ATCC 35734™) (American Type Culture Collection (ATCC) Manassas, VA, USA)and a recombinant *M. bovis* BCG harboring a pMN437 plasmid for expression of Green Fluorescent Protein (rBCG-GFP) [76], were kindly supplied by Prof Elsa Anes (FFUL). The bacterial cell culture reagents were purchased from Difco, Franklin Lakes, New Jersey, USA. Both *M. bovis* BCG Pasteur and rBCG-GFP cultures were grown on Middlebrook’s 7H9 broth Medium supplemented with 5% (*v*/*v*) OADC (oleic acid, albumin, dextrose and catalase supplement) at 37 °C/5% CO_2_.

The THP1 cells (ATCC TIB-202™) a human monocyte cell line was obtained from (ATCC, USA). All animal cell culture reagents were purchased from Invitrogen (Paisley, UK). Phorbol myristate acetate (PMA), 3-(4,5-dimethyl-2-thiazolyl)-2,5-diphenyl-2*H*-tetrazolium bromide (MTT), dimethylsulfoxide (DMSO) were all from Sigma-Aldrich (Dorset, UK).

### 3.2. Preparation of Polymeric Microparticles

Polymeric microparticles were initially prepared using modifications of previously described ionic cross-linking methods [30,31,32,33,34], by high speed stirring at room temperature and without organic solvents—Methods (I) and (II). Alginate and chitosan were dissolved in ultra-purified water.

**Figure 9:**
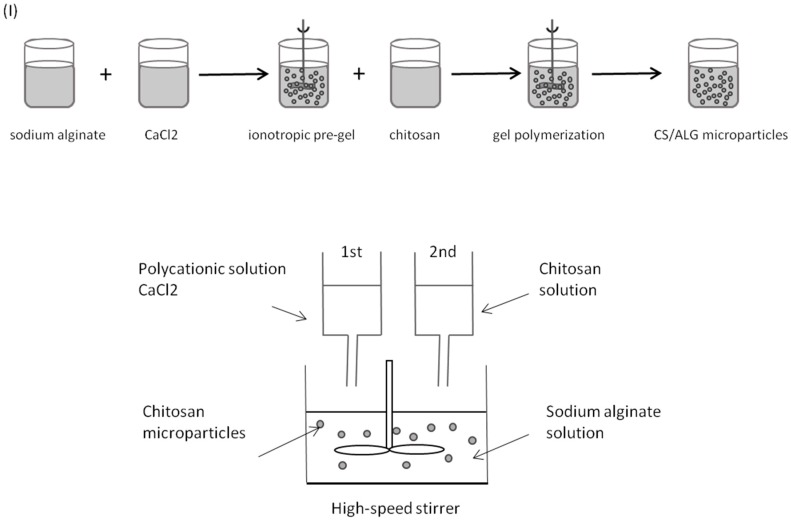
Microparticles formation by alginate ionotropic pre-gelation with CaCl_2_ followed by chitosan addition (adapted from [33]).

In Method (I), polymeric microparticles were prepared via formation of an alginate ionotropic pre-gel, by allowing sodium alginate solution to react with calcium chloride prior to chitosan addition. (Figure 9). Briefly, a 7.5 mL aliquot of 18 mM calcium chloride solution was added drop wise into a beaker containing 117.5 mL of a 0.6 mg/mL sodium alginate solution, and stirred for 60 min under 600 rpm, to provide an alginate pre-gel. Then, 25.0 mL of a 0.7 mg/mL chitosan solution was added drop wise into the pre-gel and stirred over 90 min, giving a final alginate and chitosan concentration of 0.5 mg/mL and 0.1 mg/mL, respectively (alginate: chitosan mass ratio 4.23:1). A colloidal dispersion formed upon polycationic chitosan addition.

**Figure 10:**
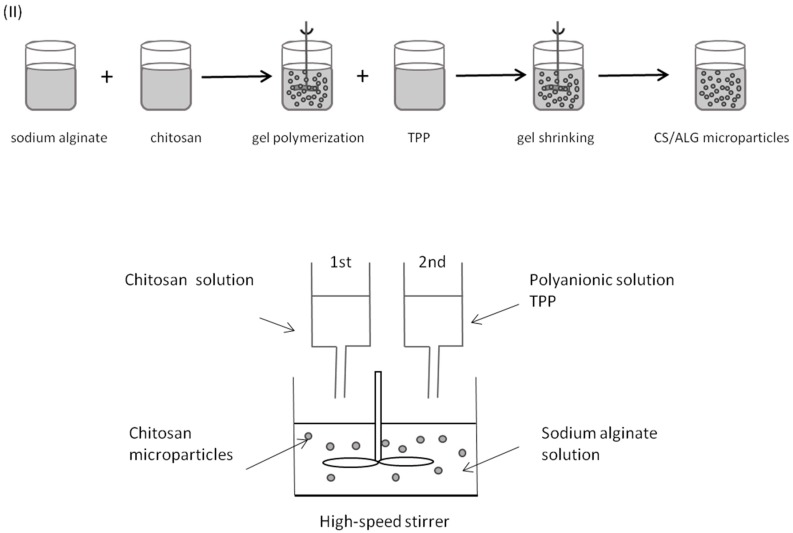
Microparticles formation by chitosan gel matrix formation with sodium alginate followed by TPP addition (adapted from [32,34]).

In formulation Method (II), polymeric microparticles were prepared by allowing chitosan and sodium alginate to polymerize, by means of ionic interaction between positively charged amine groups of chitosan and negatively charged carboxyl groups of alginate, prior to TPP addition (Figure 10). Briefly, a 5.0 mL aliquot of 1.0 mg/mL chitosan were added drop wise into a beaker containing volume ranging from 0.1 to 5.0 mL of 1.0 mg/mL of sodium alginate solution, followed by dropwise addition of 1.0 mL of 1.0 mg/mL TPP under high-speed stirring at 600 rpm for 120–150 min. Alginate: chitosan ratios ranged from 0.02:1 to 1:1 (*w*:*w*).

Preliminary experiments with three replicates were designed in order to investigate the appropriate concentration range for chitosan, alginate and sodium tripolyphosphate, according to a previously described method [32] with modifications (Table 8). The purpose was to identify the impact of the key components of the polyelectrolyte matrix, such as different pH values and polymers mass ratio, on parameters such as particle size and zeta potential. Therefore, high, medium and low molecular weight chitosan, and TPP, were used and their volume was kept constant (5.0 mL and 2.0 mL, respectively), while high, medium and low viscosity alginate was used in increasing volumes.

High shear homogenization (ultra-turrax T10basic at 11,400 rpm, IKA-Labortechnik, Staufen, Germany) and sonication (Branson Sonifier 250, equipped with a 3 mm microtip probe, BRANSON Ultrasonics Corporation, Danbury, CT, USA) were assessed as potential alternatives to high speed stirring to prepare microparticles. The techniques were evaluated considering physical stability of microparticle suspensions (general aspect, formation of aggregates) and microparticles characteristics, such as size distribution and surface charge. Microparticle preparation was in accordance with above described formulation Method (I) and Method (II), with modifications. Briefly, in accordance with previously described formulation Method (I), 0.75 mL of 18 mM calcium chloride were added to 11.75 mL of 0.6 mg/mL sodium alginate, and either homogenized for 3 min in ultra-turrax or sonicated for 3 min at 20% output intensity, prior to 2.5 mL of 0.7 mg/mL chitosan addition, and subsequent homogenization or sonication as described. As for Method (II), 5.0 mL of 1.0 mg/mL chitosan were added to 0.1–5.0 mL of 1.0 mg/mL of sodium alginate, followed by addition of 1 mL of 2.0 mg/mL TPP, and either homogenized for 3 min in ultra-turrax or sonicated for 3 min at 20% output intensity.

**Figure 11:**
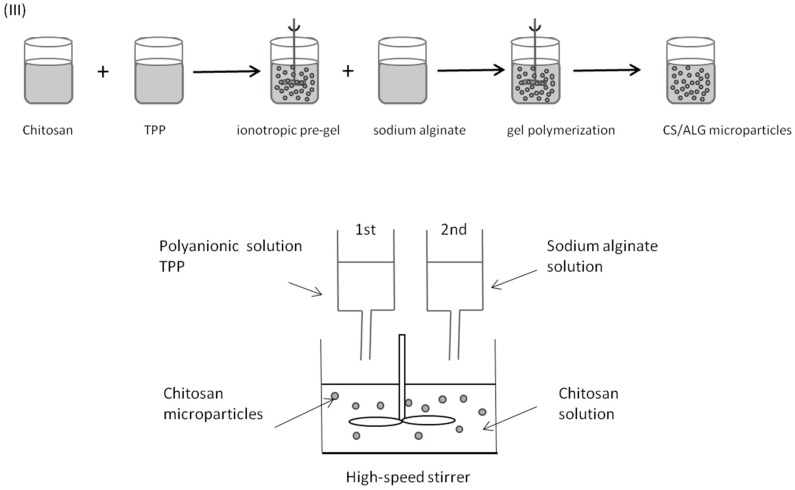
Microparticles formation by chitosan precipitation with TPP followed by alginate addition.

The results obtained during formulation optimization studies led us towards the rejection of Method (I) and the development of a different preparation method—Method (III). In Method (III), polymeric microparticles were prepared by inducing the pre-gelation of chitosan with TPP, followed by alginate coating (Figure 11). Briefly, alginate and chitosan were dissolved in ultra-purified water. Microparticles were formed by the dropwise addition of a 1.0 mL aliquot of 2.0 mg/mL TPP into a beaker containing 5.0 mL of 1.0 mg/mL chitosan solution, followed by polyanionic cross linking with volumes ranging from 0.1 mL to 5.0 mL of 1.0 mg/mL sodium alginate solution, also added drop wise, under high-speed stirring at 600 rpm for 120–150 min.

Additional characterization studies were conducted with Methods (II) and (III) to identify the best experimental conditions to obtain microparticles of intended size distribution and surface charge. In these studies, microparticles were prepared with sodium alginate of different quality specifications, namely, viscosity and guluronic acid monomers content (G-content, %), and the influence of pH value variations in microparticle size distribution and surface charge was determined. During pH value studies, a wide range of chitosan- and sodium alginate-solution pH value was assessed (pH from 3.3 to 7.6), while alginate (Protanal™ LF 10/60) concentration was kept constant at 0.1%. Modifications to the formulation methods were introduced along the optimization process accordingly to the conclusions yielded by the obtained results during preliminary studies. Regarding future lyophilisation of microparticles, some preliminary studies using two concentrations (5% and 10% *w*/*v*) of three different cryoprotectors (sucrose, glucose, and trehalose) were also conducted.

### 3.3. BCG Studies

#### 3.3.1. BCG Single Cell Suspension

Bacterial cultures in exponential growth phase (after 7 days) were pelleted at 4000 rpm (1559× *g*) at 4 °C for 15 min, washed twice in sterile 10 mM PBS pH 7.4 and re-suspended in appropriate medium. The suspension was kept on the bench for 5 min, to allow the decantation of the large clumps of bacteria. Clumps of bacteria were removed by ultrasonic treatment of bacteria suspensions in an ultrasonic water bath for 15 min. For bacteria remaining in clumps, the complete volume of the suspension was collected and pressed through a 21 gauge needle against the syringe tube wall for 10 times, in order to get individualized bacilli.

Single cell suspension was confirmed by phase contrast microscopy or in a fluorescence microscope (for rBCG-GFP). In the absence of clumps, the OD of the suspension at λ = 600 nm was adjusted to 0.1 (we assumed that 0.1 OD_600_ corresponds to 1 × 10^7^ bacteria per mL [76]). This was later confirmed by methylene blue staining and haematocytometer count (for BCG Pasteur) and CFU counts after bacterial suspension plating, in order to more accurately determine the total viable number of bacteria, as this can vary immensely as a function of the growth medium composition of mycobacteria, and also due to a potential growth inhibition effect of chitosan [77,78,79,80,81].

#### 3.3.2. Surface Charge Characterization

In order to modify the physicochemical properties of BCG, monodispersed bacteria were suspended in different concentrations of chitosan and encapsulated or adsorbed into different formulations of microparticles. Then, size distribution and surface charge were determined. These studies were performed for both BCG Pasteur and rBCG-GFP strains. Inactivation studies of both strains were also conducted to assess the effect of viability loss in BCG surface characteristics, and to perform the further characterisation studies in safety. Mycobacteria were submitted to heat inactivation, by submersion of 15 mL falcon containing bacterial suspensions in a water bath preheated and maintained at 80 °C for 15 min [82]. The efficacy of inactivation methods was determined by viability checks, as follows: 100 µL of the heat killed suspension was used to inoculate each of two plates with solid agar Middlebrook 7H10 medium supplemented with 5% OADC and incubated at 37 °C in 5% CO_2_ atmosphere for 3 weeks.

#### 3.3.3. Microencapsulation of BCG

BCG-loaded microparticles were prepared by addition of 1.0 mL of whole live attenuated BCG bacillus monodispersed in NaCl 0.9% (range, 1–2 × 10^8^ CFUs/mL) to 5.0 mL of 1.0 mg/mL chitosan solution. Next, 1.0 mL of 2.0 mg/mL TPP was added drop wise to chitosan-suspended BCG, followed by drop wise addition of 4.0 to 5.0 mL of 1.0 mg/mL sodium alginate solution to the mixture. Final concentrations of prepared microparticles ranged from 6 to 8 log10 CFUs/mL and from 0.42 to 0.45 mg/mL of chitosan.

#### 3.3.4. BCG Cell Viability

In order to assess BCG viability, a colony-forming units (CFUs) assay was used to count bacteria of both strains (Pasteur and GFP) able to produce colonies in agar Middlebrook 7H10 medium supplemented with OADC (widely used to cultivate and access the CFUs in the case of slow growers such as *M. tuberculosis* and *M. bovis* BCG [77]). Briefly, aliquots of BCG suspended in 0.25 mg/mL chitosan of medium MW and BCG-loaded chitosan-alginate microparticles were seeded in appropriate inoculation medium to determine the effects of processing conditions on cell viability. The samples were maintained at 4 °C for approximately four months (15 weeks), and plates were inoculated with samples for cell count at regular time points. After three weeks of incubation at 37 °C and 5% CO_2_, colonies were counted to determine CFUs.

### 3.4. Characterization of Microparticles

#### 3.4.1. Size Distribution, Surface Charge and Morphology

The microparticles were assessed according to size distribution and surface charge (zeta potential), by laser diffraction and electrophoretic mobility, using a Mastersizer 2000 and a Malvern Zetasizer, respectively (Malvern Instruments, Worcestershire, UK). For particle size analysis, each sample was diluted with filtered purified water to the appropriate concentration to yield 10% obscurity limit. Each analysis was carried out in triplicate at 25 °C. Results were expressed in terms of mean diameter and span (Span = d (0.9) − d (0.1)/d (0.5)). Size distribution is characterized using the d0.5 parameter (diameter for which 50% of the distribution falls below) and the span parameter (width of particle size distribution). For the determination of the electrophoretic mobility, samples were diluted with filtered purified water. The mean values were obtained from the analysis of three different batches, each of them measured three times. Morphological examination of microparticles was performed by microscopy. The ImageJ software, 1.44p version (National Institutes of Health, Bethesda, MA, USA) was used to perform image analysis.

#### 3.4.2. Production Yield

The production yield (YP) (Equation (1)) of the microparticles was determined using an indirect method based on the quantification of the chitosan concentration initially used in the formulation, and that found in the supernatant of the final microparticle suspension as previously published [29]. The method of quantification is based on a colorimetric reaction between amine groups of chitosan and the dye Cibacron brilliant red 3B-A [30].

(1) $$\text{CS yield }\left(\%\right)=\frac{\left[CS\right]total-\left[CS\right]supernantant}{\left[CS\right]total}\;\times \;100$$

#### 3.4.3. Fourier Transform Infrared Spectroscopy (FT-IR) Analysis

Preliminary information on chemical nature of the chitosan-alginate microparticles was collected using FT-IR analysis in an IRAffinity-1 (Shimadzu Corporation, Kyoto, Japan) spectrophotometer. The FT-IR measurements were made directly in the dried microparticles, which were previously lyophilised, and all powder raw materials namely chitosan and alginate, gently mixed with approximately 300 mg of micronized KBr powder and compressed into discs at a force of 10 kN for 1 min using a manual tablet presser (Perkin Elmer, Norwalk, CA, USA). All spectra were recorded at room temperature at the resolution of 4 cm^−1^ and 50-times scanning, between 4000 and 500 cm^−1^ [83].

#### 3.4.4. Encapsulation Efficiency

Encapsulation efficiency (EE, %) was determined by cell count number using a haemocytometer (Neubauer chamber Bürker). The encapsulation efficiency is expressed as the percentage of BCG entrapped/adsorbed in microparticles reported to initial amount of cells in suspension (Equation (2)).

(2) $$\text{Encapsulation efficiency }\left(\%\right)=\frac{Total\;cells-Free\;cells}{Total\;cells}\;\times \;100$$

### 3.5. In Vitro Cell Viability (MTT Assay)

Animal cell viability was assessed using general cell viability endpoint MTT as previously described with some modification [30,73]. Briefly, THP-1 cells (grown in RPMI 1640 supplemented with 10% FBS, penicillin and streptomycin,) were seeded onto 96 well cultures dishes at a density of 5 × 10^5^ cells/mL and treated for 72 h with 20 nM PMA in order to differentiate into macrophage, and medium exchanged and incubated for one more day. Cells were then incubated for 72 h at 37 °C with different concentrations of CS and ALG solutions and BCG loaded and empty formulations. Controls consisted of cells incubated with only culture medium. Each sample concentration was tested in triplicate in a single experiment, which was repeated at least 3 times.

After the incubation time, culture medium was replaced with culture medium containing 0.5 mg/mL of MTT and incubated for 3 h at 37 °C. The medium was removed after 3 h and the intracellular formazan crystals were solubilised and extracted with dimethylsulfoxide (DMSO). After 15 min at room temperature, the absorbance of the extracted solution was measured at 570 nm in a microplate reader (Infinite M200, Tecan, Männedorf, Switzerland). The percentage of cell viability was determined for each concentration of tested sample according to Equation (3), where *Abs test* is the absorbance value obtained for cells treated with samples, and *Abs control* is the absorbance value obtained for cells incubated with culture medium.

(3) $$\text{Cell viability }\left(\text{\% of Control}\right)=\frac{Abs\;test}{Abs\;control}\;\times \;100$$

### 3.6. Statistical Analysis

Data were subjected to ANOVA for analysis of statistical significance, and a *p* value of <0.05 was considered to be significant. Unless stated otherwise, results are expressed as mean values ± standard deviation (SD). The analysis was carried out using GraphPad Prism v. 5.02 (GraphPad Software, La Jolla, CA, USA).

## 4. Conclusions

During these formulation studies, it was possible to optimize the preparation method for BCG-loaded chitosan-alginate microparticles with reproducible size distribution, encapsulation efficiency and yield of preparation. Particle size and size distribution uniformity were considered to be critical aspects throughout the formulation studies. These parameters are influenced by several experimental conditions, such as the properties of the used polymers, antigen type (whole live bacteria represent additional challenges regarding cell viability maintenance during formulation); type, speed and duration of homogenization, polymer/polymer and polymer/complexation agent mass ratios, and the relationship between the pH values of the different polymers.

In this study, biodegradable and biocompatible polymers (chitosan and sodium alginate), as well as two strains of *Mycobacterium bovis* BCG (BCG Pasteur, clinically available vaccine; and rBCG-GFP), were used. The number of variables that could be optimized was reduced throughout the formulation development. Essentially, the optimization of the preparation method relied on the identification of the best polymeric compositions and identification of the crucial steps in the ionic gelation methods that were determinant for particle size distribution and surface charge.

It was possible to observe that, for chitosan-alginate microparticles, size distribution was mainly influenced by the molecular weight of the used polymers and by the type of polymer blends. On the contrary, particle surface charge was mainly influenced by polymer to polymer mass ratio due to the possibility of particle aggregation. By simply suspending BCG in chitosan, it was possible to tune BCG physicochemical properties, namely surface charge.

Additionally, the encapsulation of monodispersed whole live BCG bacilli into microparticles was of paramount importance since it could directly influence BCG cell viability and particle size and surface charge. It was possible to develop a reproducible method for microencapsulation of whole live bacteria using only mild conditions, through ionic cross-linking, with good production yield and encapsulation efficiency, while maintaining cell viability and assuring the biocompatibility of the developed microparticulate delivery system. The microencapsulation of BCG had no considerable effect on particles key features (*i.e.*, size distribution, surface charge, morphology). However, the formulation method and, to a minor extent, the concentrations of BCG used, proved to be crucial in achieving high encapsulation efficiency values.

Regarding particle surface charge, it was possible to demonstrate that the addition order of the polymers was crucial to obtaining microparticles of either electronegative or electropositive surface charge, as follows: Method (I) produced negatively charged particles, as chitosan droplets were imprisoned in a previously formed, and stoichiometric predominant, alginate matrix); Method (II) allowed the preparation of positively to negatively charged particles, depending on the polymer mass ratio (Figure 2) or polymer specifications such as alginate G-content (Figure 3); and Method (III) allowed the preparation of negatively charged particles, as alginate probably coated previously formed chitosan particles. This was clearly shown with the empty particles (Table 1, Table 3 and Table 4). As expected, due to the negative surface charge of BCG bacilli, BCG encapsulation led to minor modifications of the net charge at the particle surface (as depicted in Table 7), probably due to interference with the polymer arrangement.

In conclusion, a whole, live attenuated, cell-based particulate delivery system was developed for mucosal immunization purposes. Further characterization of these formulations in terms of *in vitro* cellular interaction with macrophages and *in vivo* study following intranasal immunization in mice is ongoing.

## Figures and Tables

**Table 1:** Particle size distribution (mean diameter and span) and surface charge (zeta potential) of microparticles on the preparation day.

Formulation	ALG:CS Mass Ratio (*w*/*w*)	Chitosan MW	Particle Size, d0.5 (µm)	Span	Zeta Potential (mV)	Production Yield (%)
F0		Low	18.5 ± 0.7	1.4 ± 0.0	−20.8 ± 7.9	n.d.
	4.23:1	Medium	260.5 ± 41.3	4.3 ± 0.6	−26.7 ± 4.9	n.d.
		High	68.1 ± 10.6	5.2 ± 2.9	−17.9 ± 6.8	n.d.
F11		Low	37.1 ± 0.7	9.5 ± 0.5	+34.0 ± 0.5	n.d.
	0.4:1	Medium	144.4 ± 5.1	3.0 ± 0.1	+47.1 ± 1.7	n.d.
		High	107.5 ± 10.1	4.1 ± 0.9	+30.4 ± 1.4	n.d.
F12		Low	39.3 ± 2.0	2.6 ± 0.1	+26.7 ± 1.1	n.d.
	0.6:1	Medium	94.7 ± 3.2	3.7 ± 0.2	+30.9 ± 1.1	n.d.
		High	65.9 ± 4.4	4.1 ± 0.7	+25.5 ± 0.5	n.d.
F13		Low	33.8 ± 0.9	2.6 ± 0.1	+22.7 ± 1.6	83.6 ± 0.0
	0.8:1	Medium	23.9 ± 0.6	2.7 ± 0.3	+25.6 ± 1.8	n.d.
		High	51.3 ± 1.8	3.3 ± 0.1	−0.2 ± 0.8	n.d.
F14		Low	25.9 ± 0.7	1.7 ± 0.1	+16.2 ± 0.6	36.8 ± 0.0
	1:1	Medium	139.0 ± 8.5	3.2 ± 0.1	−10.9 ± 1.6	n.d.
		High	80.6 ± 3.8	4.9 ± 0.2	−25.7 ± 3.3	n.d.

F0, microparticles obtained by Method (I) via alginate ionotropic pre-gelation with CaCl_2_ followed by chitosan addition; F11-F14, microparticles, obtained by Method (II) via chitosan pre-gelation with alginate, followed by TPP (pH 9.0) addition. The pH of alginate and chitosan solutions was initially set to 4.9 and 4.6, respectively. Microparticle size is characterized using the size distribution parameters d0.1, d0.5 and d0.9 (diameter for which 10%, 50% and 90% of the size distribution falls below, respectively) and span (width of particle size distribution, according to the formula (d0.1 − d0.9)/d0.5). Results are expressed as mean and standard deviation (*n*≥3). n.d., not determined.

**Table 2:** Size distribution of polymeric microparticles prepared by high-speed homogenization and ultrasonication, and yield of production.

Formulation_Chitosan MW	High-Speed Homogenization			Ultrasonication		
	Particle Size, d0.5 (µm)	Span	Production Yield (%)	Particle Size, d0.5 (µm)	Span	Production Yield (%)
F0_Low	34.5 ± 1.8	6.7 ± 0.5	n.d.	67.8 ± 10.6	5.6 ± 0.9	n.d.
F0_Medium	47.8 ± 2.2	4.1 ± 0.4	n.d.	11.5 ± 3.2	8.8 ± 1.3	n.d.
F0_High	91.8 ± 1.6	3.4 ± 0.1	n.d.	69.5 ± 9.9	6.5 ± 0.8	n.d.
F12_Low	30.2 ± 0.3	3.0 ± 0.0	n.d.	-	-	n.d.
F13_Low	20.4 ± 0.2	2.6 ± 0.0	n.d.	10.8 ± 0.6	6.7 ± 1.9	72.2 ± 0.0
F13_Medium	65.6 ± 1.7	3.5 ± 0.1	n.d.	11.8 ± 0.0	1.6 ± 0.0	97.3 ± 0.9
F13_High	52.9 ± 1.1	2.8 ± 0.0	n.d.	11.2 ± 0.4	4.7 ± 2.4	103.4 ± 2.6
F14_Low	25.2 ± 0.3	3.0 ± 0.0	53.8 ± 0.0	14.4 ± 0.3	1.2 ± 0.0	60.1 ± 10.7

F0, microparticles obtained by Method (I) with modifications, via alginate ionotropic pre-gelation with CaCl_2_ followed by chitosan addition; F12–F14, microparticles obtained by Method (II) with modifications, via chitosan pre-gelation with alginate followed by precipitation with 2 mg/mL TPP (pH 9.0), with ALG/CS mass ratios ranging from 0.6:1 to 1:1. The pH of alginate and chitosan solutions was initially set to 4.9 and 4.6, respectively. MW, Molecular weight; n.d., not determined.

**Table 3:** Size distribution and zeta potential of microparticles prepared by homogenization in an ultrasound water-bath, with increasing homogenization times.

Time (min)	F13_Medium			F14_Low		
	Particle Size, d0.5 (µm)	Span	Zeta Potential (mV)	Particle Size, d0.5 (µm)	Span	Zeta Potential (mV)
0	16.3 ± 0.1	2.0 ± 0.2	−49.8 ± 0.7	12.5 ± 0.2	1.8 ± 0.4	−14.9 ± 0.2
4	19.4 ± 0.6	2.4 ± 0.2	−29.6 ± 1.2	15.7 ± 0.2	1.9 ± 0.2	−19.5 ± 0.7
20	12.6 ± 0.1	2.0 ± 0.2	+12.1 ± 0.9	21.0 ± 0.3	1.6 ± 0.0	−14.1 ± 0.5

F13_Medium, microparticles of 0.8:1 ALG/CS mass ratio prepared with medium molecular weight chitosan and medium viscosity alginate Protanal™; F14_Low, microparticles of 1:1 ALG/CS mass ratio that were prepared with low molecular weight chitosan and low viscosity alginate Protanal™. All microparticles obtained via chitosan precipitation with TPP (pH 9.0) followed by addition of alginate (adapted from *Method III*). The pH of alginate and chitosan solutions was initially set to 4.9 and 4.6, respectively. *Medium* and *Low* refers to chitosan molecular weight and to alginate viscosity.

**Table 4:** Effect of pH on particle size distribution, particle surface charge, and yield of production of microparticles prepared with alginate to chitosan mass ratio of 1:1 (F14_Low).

ALG pH	CS pH	F14 pH	Particle Size, d0.5 (µm)		Span	Zeta Potential (mV)		Production Yield * (%)	Aggregates
3.0	3.0	3.3	44.2	±0.9	1.5 ± 0.0	+8.7	±0.1	6.4%	Yes
4.0	3.0	3.6	31.3	±0.3	31.0 ± 16.3	+3.7	±0.0	12.5%	Yes
5.0	3.0	3.9	16.1	±0.1	1.3 ± 0.0	−3.5	±0.0	11.7%	
6.4	3.0	4.0	15.2	±0.2	1.4 ± 0.1	−4.6	±0.0	17.8%	
7.0	3.0	4.0	18.0	±0.4	1.3 ± 0.0	−2.3	±0.0	n.d.	
3.0	4.0	4.1	43.8	±1.0	1.5 ± 0.0	+1.8	±0.0	16.9%	Yes
4.0	4.0	4.3	19.0	±0.8	1.7 ± 0.0	−4.9	±0.0	11.7%	Yes
5.0	4.0	4.5	13.0	±0.3	2.0 ± 0.1	−7.3	±0.1	13.6%	
6.4	4.0	4.6	13.2	±0.7	1.7 ± 0.0	−6.2	±0.1	11.9%	
7.0	4.0	4.6	15.8	±0.4	1.8 ± 0.0	−13.4	±0.1	18.3%	
3.0	5.0	4.9	12.9	±0.5	6.3 ± 1.2	−16.3	±0.0	10.4%	
4.0	5.0	5.0	12.1	±0.4	3.1 ± 0.5	−14.8	±0.0	11.4%	
5.0	5.0	5.3	11.4	±0.3	3.6 ± 0.5	−18.6	±0.1	6.7%	
6.4	5.0	5.4	10.9	±0.4	2.7 ± 0.4	−18.9	±0.1	11.4%	
7.0	5.0	5.4	11.8	±0.3	2.8 ± 0.4	−17.8	±0.1	10.6%	
3.0	6.0	5.7	11.5	±0.2	5.2 ± 0.3	−22.4	±0.1	8.3%	
4.0	6.0	6.3	10.0	±0.7	7.1 ± 0.8	−23.7	±0.2	14.2%	
5.0	6.0	7.2	11.9	±0.3	4.9 ± 0.3	−26.3	±0.1	14.6%	
6.4	6.0	7.5	11.4	±0.2	3.3 ± 0.4	−25.7	±0.1	n.d.	
7.0	6.0	7.6	13.8	±0.3	2.3 ± 0.0	−21.8	±0.2	n.d.	

F14, microparticles obtained using formulation Method (III) with low molecular weight/92% deacetylation degree chitosan (CS), and low viscosity / high-G sodium alginate (ALG) (Protanal™ LF 10/60). Results are expressed as mean and standard deviation (*n* ≥ 3). *Production yield was determined using gravimetric determination of particles mass following lyophilisation, and it is expressed as mass percentage (*w*/*w*), referred to particles theoretical mass; n.d., not determined.

**Table 5:** Characterization of plain chitosan-alginate microparticles (formulation “F14_Low”) batches without (batch A) and with (batches B to H) cryoprotectants addition (sucrose, glucose, or trehalose), on production day and following freeze-drying.

Batches	Cryoprotectant % (*w*/*v*)	ALG:CS:Cryoprotectant Mass Ratio (*w*/*w*)	Before Freeze-Drying			After Freeze-Drying	
			Particle Size (µm)		Zeta Potential (mV)	Particle Size (µm)	
			d0.5	Span		d0.5	Span
A	-	1:1:0	13.3 ± 1.0	3.1 ± 2.3	−19.5 ± 0.7	170.6 ± 50.8	2.6 ± 0.3
B	Sucrose 5%	1:1:120	13.6 ± 0.2	1.8 ± 0.5	+14.9 ± 0.3	93.5 ± 15.6	2.3 ± 0.1
C	Sucrose 10%	1:1:240	13.2 ± 1.1	1.4 ± 0.1	n.d.	65.5 ± 7.3	2.0 ± 0.2
D	Glucose 5%	1:1:120	12.9 ± 0.0	1.8 ± 0.0	n.d.	54.1 ± 9.9	1.9 ± 0.3
E	Glucose 10%	1:1:240	13.5 ± 2.0	2.0 ± 0.7	+11.6 ± 1.2	47.1 ± 8.3	2.4 ± 0.2
F	Trehalose 5%	1:1:120	13.2 ± 0.4	1.8 ± 0.3	+13.3 ± 0.5	81.9 ± 15.5	2.0 ± 0.1
G	Trehalose 10%	1:1:240	12.1 ± 0.6	1.4 ± 0.1	n.d.	63.7 ± 4.5	2.0 ± 0.3

The pH of alginate, chitosan and TPP solutions was initially set to 6.7, 4.1 and 9.0, respectively. Results are expressed as mean and standard deviation (*n* ≥ 3); n.d., not determined.

**Table 6:** Surface charge of inactivated *Mycobacterium bovis* BCG (strains Pasteur and rBCG-GFP) bacilli suspended in different media. Results are presented as mean ± SD (*n* = 3).

Inactivation Method	Medium	Zeta Potential (mV)	
		BCG Pasteur	rBCG-GFP
Temperature (80 °C, 15′)	H_2_O	−39.3 ± 1.0	−32.6 ± 1.0
	Cell culture medium	−27.9 ± 2.7	−21.2 ± 2.5
	10 mM PBS	−20.4 ± 1.5	−13.7 ± 1.4
	0.9% NaCl	−29.9 ± 11.5	−23.1 ± 11.0
	0.025% low MW chitosan	+83.9 ± 3.5	+90.6 ± 3.5
	0.1% low MW chitosan	+85.7 ± 12.1	+92.4 ± 11.9

**Table 7:** Characterization of batches of BCG-loaded “F14_Low” microparticles prepared with increasing concentrations of BCG.

Batches	Formulation Method	BCG Pasteur Load (CFU/mL)	Particle Size, d(0.5) (µm)	Span	Zeta Potential (mV)	E.E. (%)
A	II	*-*	60.9 ± 5.5	3.0 ± 5.5	14.1 ± 0.6	--
B	II	1.7×10^6^	35.3 ± 2.6	1.8 ± 0.1	10.5 ± 1.5	70.0 ± 1.6
C	II	8.3×10^6^	39.3 ± 3.9	2.8 ± 1.8	12.9 ± 2.3	74.0 ± 3.9
D	II	1.7×10^7^	41.0 ± 6.3	2.2 ± 0.1	11.8 ± 2.5	85.0 ± 4.8
E	II	3.3×10^7^	36.8 ± 2.0	2.0 ± 0.2	13.0 ± 3.9	87.0 ± 3.4
F	III	*-*	18.2 ± 1.2	3.2 ± 0.7	−3.2 ± 0.9	--
G	III	1.7×10^6^	22.2 ± 1.0	6.5 ± 2.6	−16.4 ± 2.1	--
H	III	8.3×10^6^	28.3 ± 4.0	25.5 ± 12.3	−12.7 ± 2.4	76.0 ± 1.4
I	III	1.7×10^7^	22.5 ± 2.7	4.0 ± 2.8	−13.7 ± 2.0	84.0 ± 3.4
J	III	3.3×10^7^	21.4 ± 2.4	2.5 ± 0.4	−12.2 ± 2.6	83.0 ± 10.7

**Table 8:** Values for the investigated variables during formulation studies.

Formulation	Chitosan (CS) (% *w*/*v*)	Alginate (ALG) (% *w*/*v*)	CaCl_2_* or TPP ** (% *w*/*v*)	ALG:CS Mass Ratio (*w*/*w*)
F0	0.010	0.050	0.010 *	4.23:1
F1	0.070	0.001	0.028 **	0.02:1
F2	0.069	0.003	0.028 **	0.04:1
F3	0.068	0.004	0.027 **	0.06:1
F4	0.068	0.005	0.027 **	0.08:1
F5	0.067	0.007	0.027 **	0.10:1
F6	0.066	0.008	0.026 **	0.12:1
F7	0.065	0.009	0.026 **	0.14:1
F8	0.064	0.010	0.026 **	0.16:1
F9	0.063	0.011	0.025 **	0.18:1
F10	0.063	0.013	0.025 **	0.20:1
F11	0.056	0.022	0.022 **	0.40:1
F12	0.050	0.030	0.020 **	0.60:1
F13	0.045	0.036	0.018 **	0.80:1
F14	0.042	0.042	0.017 **	1.00:1

F0—ALG/CS microparticles, obtained via alginate ionotropic pre-gelation with CaCl_2_ followed by chitosan addition—Method (I); F1–F14—CS/ALG microparticles, obtained via chitosan precipitation with TPP followed by gelation with alginate—Method (II). * CaCl_2_ ** TPP.

## Abbreviations

The following abbreviations are used in this manuscript:

ALG	Alginate
APCs	Antigen presenting cells
BCG	Bacillus Calmette-Guérin
CFU	Colony-forming unit
CS	Chitosan
DMSO	Dimethylsulfoxide
E.E.	Encapsulation efficiency
FT-IR	Fourier transform infrared spectroscopy
G	Guluronate
GFP	Green fluorescence protein
HMW	High molecular weight
HV	High viscosity
LMW	Low molecular weight
LV	Low viscosity
M	Mannuronate
MMW	Medium molecular weight
MV	Medium viscosity
MTT	3-(4,5-dimethyl-2-thiazolyl)-2,5-diphenyl-2H-tetrazolium bromide
NALT	Nasal associated lymphoid tissue
ND	Not determined
OADC	Oleic acid, albumin, dextrose and catalase supplement
OD	Optical density
PBS	Phosphate-buffered saline solution
PLGA	Poly(d,l-lactide-co-glycolide)
PLA	Poly(l-lactide)
PMA	Phorbol myristate acetate
rBCG	Recombinant BCG
THP-1	human monocyte cell line
TPP	Tripolyphosphate
US	Ultrasonication
UT	Ultraturrax
YP	Yield of production
